# Successful treatment of idiopathic tetanus using metronidazole, magnesium, and acepromazine in Hanwoo (Korean indigenous cattle) yearling bull

**DOI:** 10.3389/fvets.2023.1142316

**Published:** 2023-03-23

**Authors:** Youngjun Kim, Ji-Yeong Ku, Kichan Lee, Bo-Youn Moon, Seungmin Ha, Kyoung-Seong Choi, Jinho Park

**Affiliations:** ^1^Department of Veterinary Internal Medicine, College of Veterinary Medicine, Jeonbuk University, Iksan, Republic of Korea; ^2^Department of Animal Hospital, Hanwoo Genetic Improvement Center, NongHyup Agribusiness Group Inc., Seosan, Republic of Korea; ^3^Animal Disease Diagnostic Division, Animal and Plant Quarantine Agency, Gimcheon, Republic of Korea; ^4^Department of Animal Resource Development, Dairy Science Division, National Institute of Animal Science, Rural Development Administration, Cheonan, Republic of Korea; ^5^Department of Animal Science and Biotechnology, College of Ecology and Environmental Science, Kyungpook National University, Sangju, Republic of Korea

**Keywords:** Hanwoo (Korean indigenous cattle), idiopathic tetanus, metronidazole, magnesium, acepromazine

## Abstract

Bovine tetanus is a serious infectious disease of the central nervous system caused by the exotoxin produced by *Clostridium tetani* and is characterized by persistent tension and spasm of the rhabdomyocytes. Currently, many studies have focused on diagnosing tetanus; however, only a few studies on treatment methods have been conducted. Therefore, cattle with tetanus have been treated using symptomatic therapy. In this case, severe muscle spasticity and spasms were observed in a 9-month-old Hanwoo (Korean indigenous cattle) bull, and aspartate aminotransferase and creatine kinase levels were increased in serum biochemical tests. Clinically, bovine tetanus was strongly suspected, and metronidazole was administered orally for 5 days. To treat the intensifying bloat, a temporary rumenostomy was performed on the third day of onset, and the toxin gene (tetanospasmin) of *C. tetani* was amplified by polymerase chain reaction analysis from the collected ruminal fluid. Magnesium and sedatives (acepromazine) were administered for 7 days to treat muscle spasticity and spasms. Muscle spasticity and spasm markedly improved, and the bull stood up from the lateral recumbent position. On the 17^th^ day after onset, all tetanus-related symptoms resolved and a normal diet was started. Our findings demonstrated that treatment with metronidazole, magnesium, and acepromazine was effective in the bull with tetanus.

## Introduction

Tetanus is a serious infectious disease of the central nervous system that causes persistent tension and spasm of the rhabdomyocytes by an exotoxin produced by *Clostridium tetani* (1, 2). The route of transmission of tetanus in cattle can be divided into generalized tetanus through wounds, neonatal tetanus through umbilical cord infection, and idiopathic tetanus without apparent wounds (3–5). However, generalized tetanus mainly occurs through castration, dehorning, ear tag installation, and postpartum uterine infection (6). Subsequently, muscle spasticity and tremor in the neck region appear, the ears turn back, and the protrusion of the third eyelid is conspicuous. Additionally, eating disorders and dysphagia due to trismus have been observed, and responsiveness to external stimuli, such as light or sound, may increase. Moreover, elevated tail and hind limb spasticity are the main clinical symptoms (7–10), and secondary ruminal bloat may occur because of difficulty in the belching reflex owing to the contraction of the laryngopharynx and esophageal muscles (11). Progression of the symptoms of tetanus to a moderate or more severe condition results in a laterally recumbent position due to the rigidity of the hind limbs, which intensifies ruminal bloat owing to the inability to stand and eventually causes respiratory failure and death.

Although tetanus can be diagnosed through the clinical symptoms of the patient, laboratory tests are necessary to confirm the diagnosis. However, *C. tetani* are difficult to isolate due to the characteristics of anaerobic bacteria (12, 13). Recently, the detection of tetanospasmin in *C. tetani* using real-time polymerase chain reaction (PCR) has become possible, and it has been used to diagnose tetanus in livestock (14–16).

The classical treatment for tetanus in cattle is disinfection of the necrotic area of the wound, removal of necrotic tissue, non-oral administration of penicillin and anti-tetanus serum to remove circulating toxins, and administration of sedatives such as acepromazine or diazepam to improve muscle tone (7, 17, 18). However, the administration of an antitoxin agent to cattle is excessively expensive in clinical practice, and the administration of a sedative for muscle relaxation is also a burden on the clinical veterinarian in terms of dosage and frequency. Although many studies have been conducted on the diagnosis of tetanus, studies related to treatment are few (6, 10). As no definitive treatment for tetanus in cattle has been suggested, treatment is mainly based on symptoms. Here, we report a tetanus case that was successfully treated using magnesium and sedatives without the administration of expensive antitoxins in a Hanwoo rearing bull.

## Case presentation

**Figure 1:**
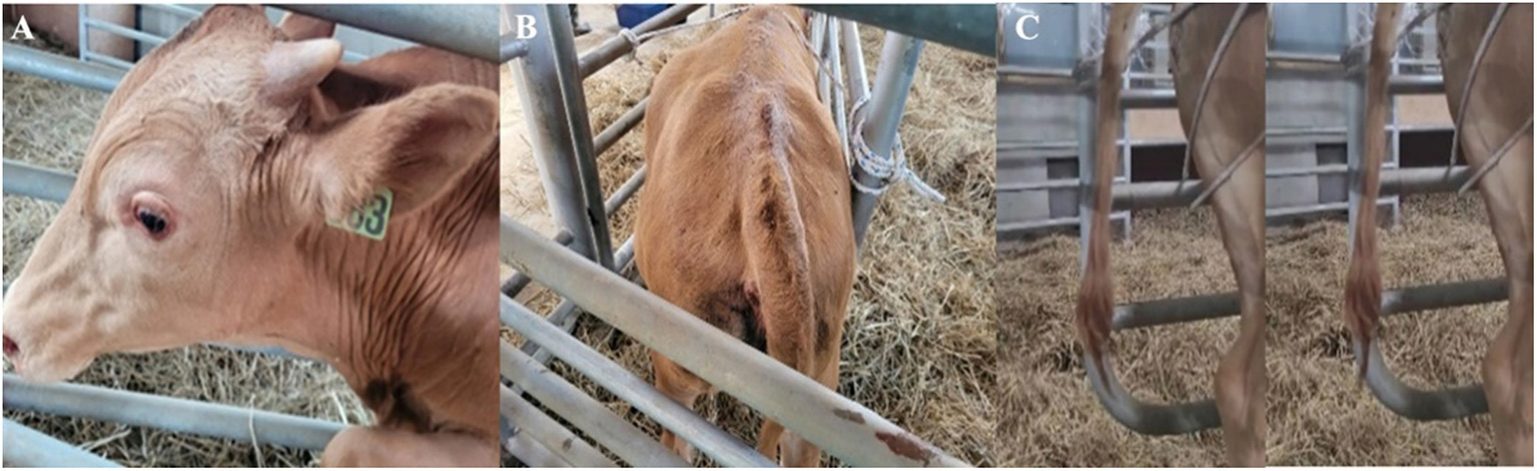
The early onset clinical symptoms of the patient with tetanus. The increase in neck muscle stiffness rendered it unable to be flexed manually. In addition, the ears were pulled back, and protrusion of the third eyelid was observed **(A)**. The coccyx is elevated and the left shoulder is bloated **(B)**. Cramps and twitching of the tail were also observed **(C)**.

A 9-month-old Hanwoo bull was found to have increased responsiveness to external stimuli, ears pulled back, and protrusion of the third eyelid (Figure 1A). At the time of onset, physical examination revealed the following: a body temperature of 38.9°C, heart rate of 92 beats per min, and respiratory rate of 61 cycles per min. The degree of spasticity of the hind limbs was insignificant, and no external wounds capable of inducing tetanus were observed. Although not confirmed, tetanus was strongly suspected, and 22,000 IU/kg of procaine penicillin (Procillin inj., Cheilbio, Korea) was intramuscularly injected; moreover, no sedatives were administered because muscle rigidity was not severe. On the second day, the spasticity of the neck muscles became severe, and spasticity of the hind limbs was noted. Mild ruminal bloat was observed with an elevation of the tail head (Figure 1B). Additionally, convulsions in the tail were conspicuously observed (Figure 1C). Therefore, typical tetanus was suspected based on these clinical symptoms.

Early onset changes in the clinical symptoms are summarized in Table 1. On the first day of outbreak on assuming a lateral recumbent position, the bull attempted to stand up on its own but failed. On the second day, the lateral recumbent position was maintained, and on the third day, the neck spasticity intensified and it was impossible for the bull to ingest feed on its own. The symptoms of muscle spasticity and convulsions gradually worsened; hence, the degree of tetanus was considered very severe.

**Table 1:** Changes in the clinical symptoms observed for 3 days after the onset of tetanus symptoms.

**Clinical signs**	**Day 0**	**Day 1**	**Day 2**
Neck stiffness	**++**	**+++**	**+++**
Prolapse of the third eyelid	**++**	**++**	**++**
Elevated tail	**–**	**+++**	**+++**
Ruminal bloat	**–**	**+**	**++**
Tail spasm	**–**	**-**	**+++**
Hind limb stiffness	**–**	**++**	**+++**
		Saw-horse posture	Lateral recumbency

**–:** Normal, **+**: Mild, **++**: Moderate, **+++**: Severe.

**Figure 2:**
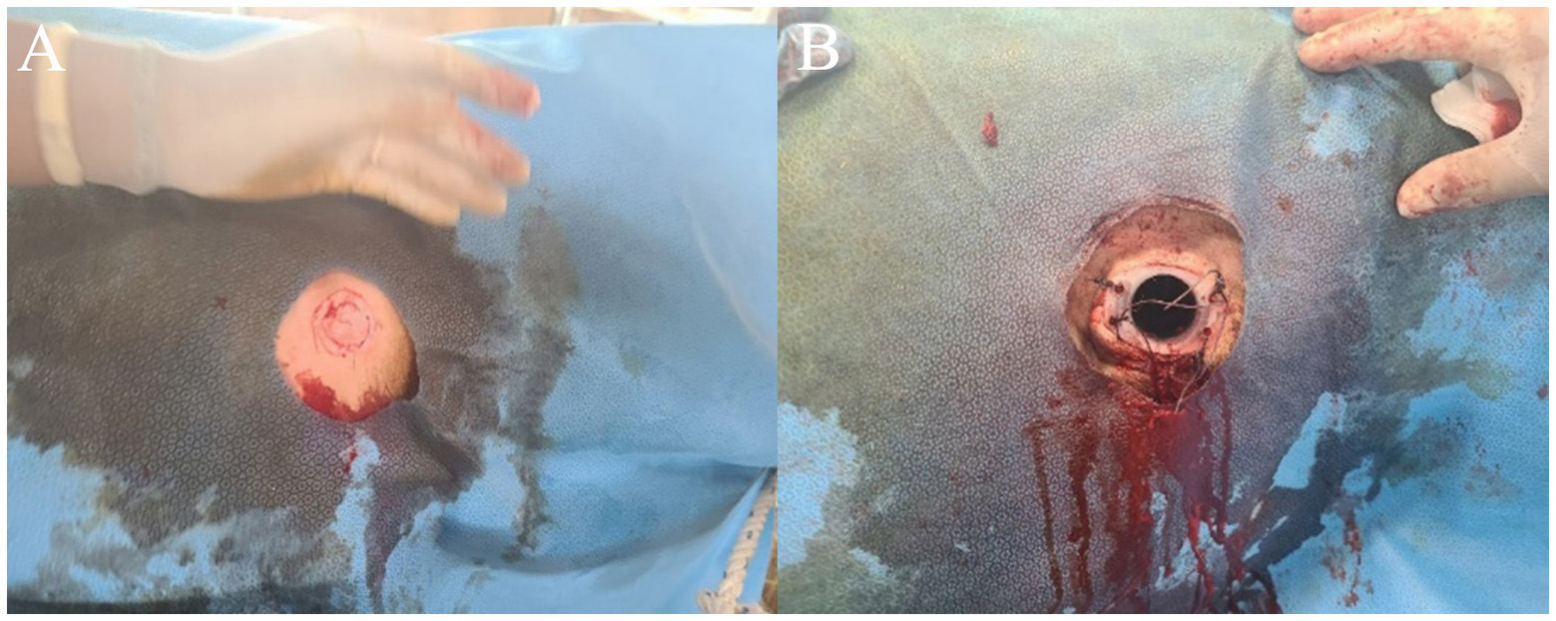
Temporary rumenostomy in a patient with tetanus **(A)**. On the third day of the onset of tetanus, a temporary rumenostomy was performed for the treatment of secondary bloat **(B)**.

On the third day of diagnosis, intensive care was started. The animal was moved to a dark place to minimize light and sound stimulation, and feed and water containers were hung at head height. Because the ruminal bloat was severe, an attempt was made to expel gas through a stomach tube; however, this procedure failed due to increased tension in the laryngopharyngeal muscles. Therefore, a temporary rumenostomy was performed. The bull in the lateral recumbent position was erected using a cow lift. Then, a circular, 4-cm-diameter incision was made in the left waist, and a rumenostomy was performed using a cannula *via* a 50 mL syringe (Figure 2A). A cover was then installed on the rumen cannula for gas release and nutrient supply to relieve ruminal bloat and was maintained for 5 days (Figure 2B). Additionally, metronidazole (Metrojyl, JSK, Korea) was orally administered at a dose of 25 mg/kg every 12 h for 5 days to treat the infection.

## Hematological and molecular analyses

**Figure 3:**
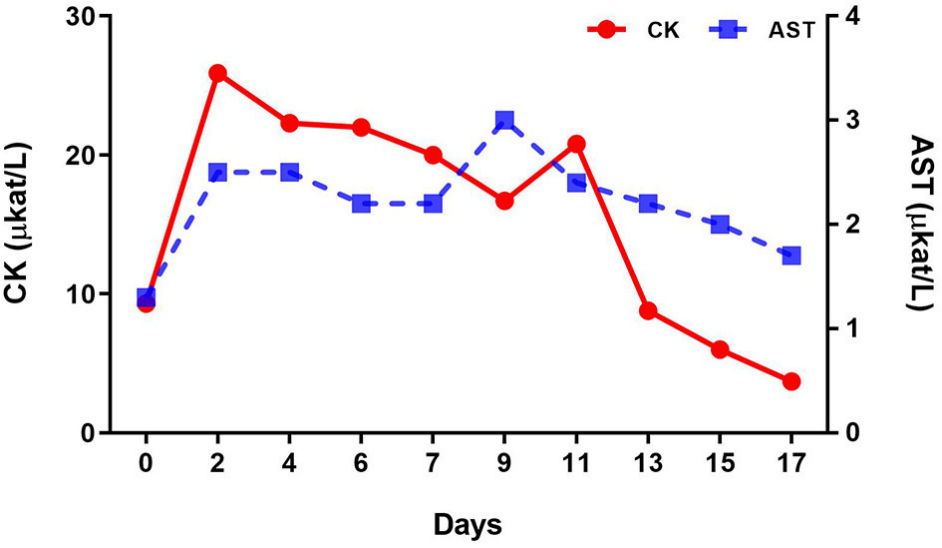
Changes in creatine kinase (CK) and aspartate aminotransferase (AST) levels after the onset of tetanus and during treatment.

Complete blood count analysis showed a red blood cell counts of 10.8 T/L and hemoglobin level of 8.5 mmol/L on the first day of diagnosis; relative erythrocytosis was confirmed secondary to dehydration. However, the white blood cell values were within the normal range. Changes in serum biochemistry test results at the time of diagnosis and after the assumption of lateral recumbent position are described in Table 2. Creatine kinase (CK) (9.3 μkat/L) and phosphorus (3.8 mmol/L) levels were increased at the time of diagnosis. After the lateral recumbent position was assumed, aspartate aminotransferase (AST) (2.5 μkat/L) and CK (25.9 μkat/L) levels were also increased, including blood urea nitrogen (4.3 mmol/L) and creatinine (159.3 μmol/L). In contrast, Figure 3 shows the changes in AST and CK levels, which are indicators of muscle damage, immediately after diagnosis until 3 weeks.

**Table 2:** Serum biochemical analysis results at the tetanus symptom onset and after assuming the lateral recumbent position.

**Assay**	**Day 0**	**Day 2**	**Normal range**
Total protein (g/L)	78	83	67–75
Albumin (g/L)	36	36	30–36
Globulin (g/L)	42	47	30–35
Total bilirubin (μmol/L)	5.1	8.5	0.2–8.5
GGT (μkat/L)	0.2	0.3	0.3–0.7
AST (μkat/L)	1.3	2.5	1.3–2.4
ALP (μkat/L)	2.4	4.7	0–8.1
CK (μkat/L)	9.3	25.9	0.7–3.5
Blood urea nitrogen (mmol/L)	5.3	9.3	7.1–10.7
Creatinine (μmol/L)	150.3	159.2	88.4–176.8
Magnesium (mmol/L)	1	0.9	0.7–1
Calcium (mmol/L)	2.9	2.5	2.4–3.1
Phosphorus (mmol/L)	3.8	2.7	1.8–2.1

**Figure 4:**
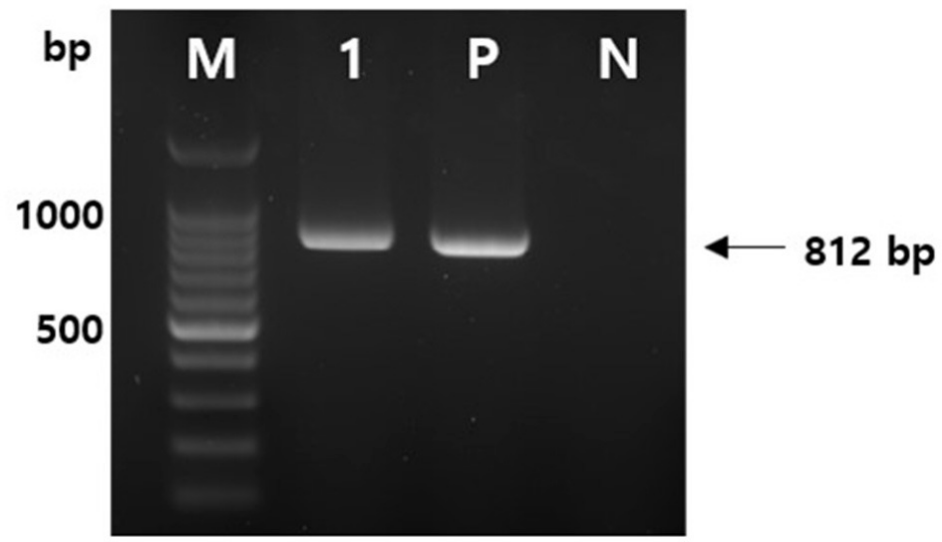
Amplification of *C. tetani* tetanospasmin (*tetX*) toxin gene by polymerase chain reaction (PCR). PCR was performed with using the DNA extracted from a single colony of ruminal contents. (M) 100 bp DNA ladder, (1) toxin gene of *C. tetani* isolate, (P) positive control, and (N) negative control.

A temporary rumenostomy was performed to collect ruminal fluid and hay in the rumen, and tetanus was detected. After the rumen contents were suspended in sterile phosphate buffered saline, the suspension was heated to 80°C for 10–15 min and then cooled to room temperature. After inoculating the pre-treated suspension on cooked meat medium, from which dissolved oxygen is removed, it was cultured at 37°C for 2 days. Suspected tetanus colonies were observed after a subculture in a blood medium and were used to amplify *C. tetani* (NF255, 5′-GCCGGAAAGGTATGAATTG-3′; NF256, 5′-TGTTGGGATCATTGCAGCTA-3′) under the following conditions: 95°C for 5 min, followed by 35 cycles at 95°C for 1 min, annealing at 52°C for 1 min, and 72°C for 2 min, followed by a final extension step at 72°C for 5 min. The amplification (812 bp) of the tetanospasmin gene, the toxin gene of *C. tetani*, was confirmed (Figure 4).

## Results

After the rumenostomy, the lateral recumbent position was reestablished, and 0.9% normal saline was infused according to the degree of dehydration (8%) to treat extensive azotemia, which was secondary to dehydration. Subsequently, 2 g of magnesium (Daihan Magnesium Injection 10%, Dai Han Pharm Co., Ltd., Korea) and 5 mg/100 kg of acepromazine (Sedaject, Samu Median Co., Ltd., Korea) were diluted in 500 mL of saline, and continuous intravenous infusion was administered for 20 min. Visually, the muscle spasticity decreased to some extent; however, no definite effect was observed. The concentration of serum magnesium before (0.9 mmol/L) and 30 min after (0.98 mmol/L) administration was not increased. After 6 h, 8 g of magnesium and 5 mg/100 kg of acepromazine were diluted in 500 mL of saline and re-infused intravenously for 20 min. Consequently, an increase in serum magnesium concentration (0.9 mmol/L before administration, 1.3 mmol/L in 30 min after administration) was observed, and the bull in the lateral recumbent position stood on its own with the improvement of muscle spasticity and convulsions. Additionally, the bull fed from the feeder at head height on its own.

**Figure 5:**
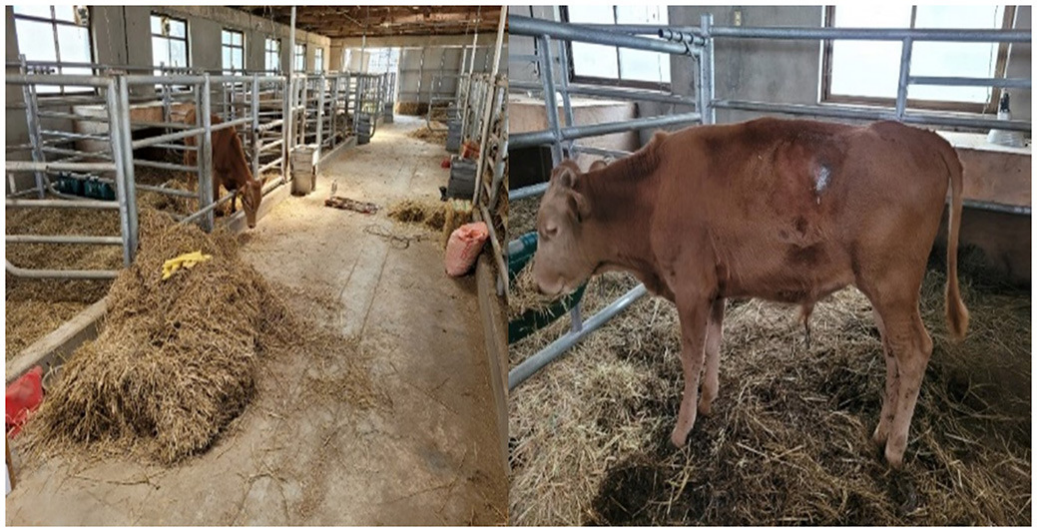
Relieved tail elevation and normal feed intake after tetanus treatment. Due to the treatment of tetanus (17^th^ day of occurrence), recovery of both the stiff hind limb and tail elevation was observed, and the patient began to freely eat food from the floor.

Magnesium and sedatives (Magnesium sulfate 6 g + acepromazine 5 mg/100 kg) were administered at intervals of 6–8 h (3 times/day) for a total of 5 days and then at intervals of 12 h (2 times/day) for 2 days. For the first 2 days, only after the administration of magnesium and acepromazine, the bull could stand up from the lateral recumbent position and feed from the feeder hung at the height of the head. However, severe salivation and difficulty in chewing were still observed, and after 2–3 h, hind limb and tail convulsions recurred along with lateral recumbency. On the sixth day of diagnosis, the spasticity of the neck muscles gradually resolved, the pulled-back ear returned to its original state, and the third eyelid protrusion was restored. However, hind limb spasticity and spasm of the tail persisted. On the seventh day, the spasticity of the hind limbs was greatly alleviated, and no more ruminal bloats occurred; thus, the site where the rumenostomy was performed was closed. However, the patient was unable to eat the bottom feed and could only feed at the height of the head. On the 11^th^ day, the spasm of the tail recovered; however, mild spasticity of the hind limb and elevation of the tail head were observed. On the 17^th^ day, all the spasticity of the hindlimb and elevation of the tail head resolved completely, and the patient began to eat the bottom feed (Figure 5). The changes in clinical symptoms observed during tetanus treatment of the patient for 3 weeks are summarized in Table 3.

**Table 3:** Changes in the clinical symptoms observed during the treatment of patient with tetanus.

**Clinical signs**	**Day 6**	**Day 7**	**Day 11**	**Day 17**
Neck stiffness	**+**	**+**	**+**	**–**
Prolapse of the third eyelid	**–**	**–**	**–**	**–**
Elevated tail	**++**	**+**	**+**	**–**
Ruminal bloat	**+**	**–**	**–**	**–**
Tail spasm	**++**	**+**	**–**	**–**
Hind limb stiffness	**++**	**+**	**+**	**–**

**–**: Normal, **+**: Mild, **++**: Moderate.

## Discussion

According to the route of transmission, tetanus is divided into generalized, neonatal and idiopathic type. In cattle, generalized tetanus is the most common, and the infection occurs through wounds. However, in this case, no special wound was found externally, and tetanus was diagnosed based on the rumen contents. Although animal species are different, only half of the horses are infected with tetanus owing to suspected infected wounds (19). Additionally, idiopathic tetanus, which occurs in rearing cattle during grazing owing to oral or gastrointestinal wounds caused by low-quality rough forage, has often been reported (3). In this case, no external wounds suggestive of infection were found, and tetanus toxin was detected in the rumen contents, suggesting that it could be classified as idiopathic tetanus.

Tetanus is a disease caused by the tetanus toxin, which is transmitted retrogradely through the nerves (20). Therefore, because the cranial somatic nerve is shorter than the limb somatic nerve, symptoms appear first in the neck and head, followed by hind limb spasticity (21). According to the authors' previous cases, tetanus symptoms temporarily appeared mainly in the neck and head in mild cases; however, in severe cases, clinical signs began in the neck and head and progressed very quickly to hind limb spasticity. In this case, it took only 24 h to assume the lateral recumbent position after the onset of clinical symptoms, and the clinical symptoms progressed very quickly.

Among the various diagnoses of the nervous system, a test for muscle tone exists. When a disorder occurs in lower motor neurons, muscles, or the cerebellum, muscle tension decreases and flaccidity occurs. However, when upper motor neurons or basal nuclei are damaged, muscle tension increases. At this time, clinicians use the term spasticity or rigidity. The former is caused by upper motor neuron damage and is characterized by the clasp-knife phenomenon, whereas the latter is caused by damage to the basal nuclei and is characterized by cogwheel rigidity. In this case, neurological examination at the time of diagnosis showed that the hind limbs could be folded to some extent if the initial resistance was passed, suggesting spasticity. Later, in the lateral recumbent position, neurological examination showed that resistance could be felt even after the initial resistance had passed, suggesting rigidity. Tetanusspasmin is inhibiting the gamma-motorneurons and is impairing the alpha-motorneurons in the spinal cord and brain stem. These are causing the muscle spasticity.

In the present case, metronidazole was used instead of penicillin as the classic treatment for tetanus to eliminate the infectious agent. Tetanus toxin infects retrogradely, irreversibly attaches to the synapse, and blocks the action of neurotransmitters such as γ-aminobutyric acid (GABA), which suppresses the excitation of nerve cells in the central nervous system, and glycin, a substance that suppresses the excitation of nerve cells in the spinal cord, causing rigidity and spasmticity. Rodrigo et al. (18) reported that penicillin, a classic treatment for tetanus, is no longer recommended because it is an antagonist of GABA and can intensify spasms. Additionally, penicillin is a beta-lactam antibiotic, and treatment could be delayed if *Staphylococcus aureus* or *Escherichia coli*, which are resistant to beta-lactam antibiotics, are present in the wound. Metronidazole is a bactericidal antibiotic for a wide spectrum of anaerobic organisms and rapidly achieves effective therapeutic concentration even though abscess cavities (22). Therefore, metronidazole (Metrojyl, JSK, Korea) was administered orally at a dose of 25 mg/kg at 12 h intervals for a total of 5 days instead of procaine penicillin, which is a classic treatment for tetanus.

Secondary ruminal bloat may occur in cattle affected by tetanus, consequently leading to death due to respiratory failure. The disturbance in the eructation reflex caused by spasms of the pharyngeal, laryngeal, and proximal esophageal muscles, due to tetanus may result in ruminal bloat. In our experience, in the bulls with tetanus, gastric tube insertion is challenging owing to pharyngeal spasms. In addition, lateral recumbency itself can intensify ruminal bloat. Thus, we believe that rumenostomy is necessary for cattle with moderate or higher clinical symptoms (23).

In general, the anti-tetanus toxin is used for the treatment of cattle with tetanus. However, a high dose of 20 IU/kg or more must be administered, which incurs a high cost of >940 USD in Korea for 500 kg cattle. Additionally, since anti-tetanus serum can only neutralize unbinding toxins, it cannot act on toxins already irreversibly attached to the neuromuscular junction. In addition, side effects, such as hepatic necrosis, have been reported in horses treated with the anti-tetanus serum. Thus, no anti-tetanus serum was administered in our case.

Traditionally, sedatives and muscle relaxants have been administered to treat rigidity and spasms caused by tetanus. This led to the administration of sedatives such as acepromazine and GABA agonists such as diazepam and phenobarbital (7). However, drugs such as diazepam are expensive due to their long-term, high-dose treatment, making them difficult to use for most industrial animals. In this case, an intravenous injection of magnesium sulfate was used instead of diazepam. Magnesium can block neuromuscular transmission and inhibit acetylcholine release from nerves to alleviate autonomic hyperactivity (24, 25). Additionally, various advantages, such as muscle relaxation, spasm relief, and palpitation control, have been found in humans (26, 27), and it is effective for treating rigidity and spasm resulting from tetanus (27, 28). According to the authors′ experiences, the use of acepromazine, a sedative alone, in Hanwoo cattle with tetanus is ineffective, and even at higher doses, it is not capable of preventing spasticity and convulsions. However, acepromazine has a satisfactory effect when used at an appropriate dose.

## Conclusion

This study describes a recovered case of tetanus in a bull in the ROK. Our data demonstrated that metronidazole, magnesium, and sedatives (acepromazine) are effective in treating bulls with tetanus.

## Data availability statement

The original contributions presented in this study are included in the article, further inquiries can be directed to the corresponding authors.

## Ethics statement

The animal study was reviewed and approved by Institutional Animal Care and Use Committee Decision No. JBNU 2021-091.

## Author contributions

YK performed all treatments and analyzed the data. J-YK analyzed the data. KL and B-YM performed PCR analysis. SH visualized all figures. YK, K-SC, and JP wrote the manuscript. All authors have read and approved the final manuscript.
